# Regulation of normal human polyrnorphonuclear leucocytes by carnitine

**DOI:** 10.1155/S0962935193000742

**Published:** 1993

**Authors:** Andrea Fattorossi, Roberto Biselli, Anna Casciaro, Sonia Tzantzoglou, Claudio de Simone

**Affiliations:** 1 Rep. Medicina Lab. Immunologia Pratica di Mare Pomezia Roma Italy; 2 Cattedra Malattie Infettive Universitá “La Sapienza” Roma Italy; 3 Cattedra Malattie Infettive Universitá di L'Aquila Italy

## Abstract

The effect of carnitine, a drug that plays an essential role in mitochondria metabolism, on some of the most important human polymorphonuclear leucocytes (PMN) activation steps including modulation of adhesion molecule density, reactive oxygen species production, and tumour necrosis factor-α (TNFα) production was investigated. The capability of carnitine in protecting PMN from deter ioration on storage was also studied. Data shows that carnitine exerts considerable effects on all PMN functions investigated. Although the ultimate effect was often donor dependent, TNFα production was exceptional in that carnitine was able to consistently reduce TNFα production in Staphylococcus aureus stimulated PMN in a clear dose-dependent fashion. It is concluded that carnitine may represent a useful active agent in situations characterized by PMN mobilization/activation.


Mediators of Inflammation 2, S37–S41 (1993)

### Regulation of normal human polymorphonuclear leucocytes by carnitine

Andrea Fattorossi,[1,CA] Roberto Biselli,[1] Anna Casciaro,[1] Sonia Tzantzoglou,[2] and Claudio de Simone[3]

[1]Rep. Medicina, Lab. Immunologia, Pratica di Mare, Pomezia, Roma, Italy;
[2]Cattedra Malattie Infettive Universitá "La Sapienza" Roma, Italy;
[3]Cattedra Malattie Infettive Universitá di L'Aquila, Italy

[CA] Corresponding Author

**THE effect of carnitine, a drug that plays an essential role in mitochondria metabolism, on some of the most important human polymorphonuclear leucocytes (PMN) activation steps including modulation of adhesion molecule density, reactive oxygen species production, and tumour necrosis factor-α (TNFα) production was investigated. The capability of carnitine in protecting PMN from deter ioration on storage was also studied. Data shows that carnitine exerts considerable effects on all PMN functions investigated. Although the ultimate effect was often donor dependent, TNFα production was exceptional in that carnitine was able to consistently reduce TNFα production in *Staphylococcus aureus* stimulated PMN in a clear dose-dependent fashion. It is concluded that carnitine may represent a useful active agent in situations characterized by PMN mobilization/activation.**

**Key words:** Activation, Carnitine, Cytokines, Polymorphonuclear leucocytes

### Introduction

L-Carnitine gamma-trimethylamino-β-hydroxybutyrate) is an essential component of cellular metabolism as it plays a pivotal role in transporting activated long-chain acyl groups across mitochondrial membrane for β-oxidation.[1] Although a large body of data is available on the involvement of carnitine in cellular metabolism in cardiac and skeletal muscle, there is much less information on the effect of carnitine on cells of the immune system, such as polymorphonuclear leucocytes (PMN). PMN serve as major effector cells in host defence and acute inflammation. A PMN leucocytosis and a PMN accumulation in tissues is a common feature of many clinical situations where PMN exert their primary role in controlling the expansion and dissemination of pathogenic microorganisms.[2]

PMN activities, such as their elevation in number, margination, and extravasation into inflammed tissues are under the control of a number of cytokines which also regulate PMN functions in the inflammatory site. PMN in turn, synthesize and release numerous immunoregulatory cytokines.[3] Thus, PMN are at the centre of a vast array of coordinated signals to which they must respond with a series of finely tuned biochemical and cellular responses to exert their defensive task. In order to become fully functioning defensive effectors, PMN undergo a series of sequentially related events, among which adhesion to the vascular endothelium via specialized adhesion molecules and transendothelial migration, destruction of pathogens via oxidative reactions, and production of cytokines with immunoregulatory functions are of paramount importance.[2,3] Since all these functions are conceivably subject to regulation by a cellular metabolism modulator such as carnitine, a series of *in vitro* assays was devised in order to study the efficiency of carnitine in these diverse steps which are instrumental for a correct PMN functioning.

### Materials and Methods

*PMN preparation:* To prevent exposure of PMN to toxic or stimulatory conditions likely to be a source of artefactual results, the use of density gradient centrifugation procedures was avoided whenever possible.[4] Thus, PMN were obtained as a total leucocyte population from peripheral blood after lysis of erythrocytes with buffered ammonium chloride. A second lysis step was performed if the cell pellet remained visibly red. PMN were then singled out from other blood cells using the electronic capabilities of a flow cytometer (see below). After lysis, PMN were resuspended in RPMI 1640 medium at a concentration of $2 \times 10^6$/ml and kept on ice until use. For experiments aimed at investigating tumour necrosis factor-α (TNFα) production by PMN, these had to be purified from peripheral blood using appropriate density gradient centrifugation, washed and resuspended in RPMI 1640 medium supplemented with 5% foetal bovine serum albumin. Non-adherent polypropylene tubes were used throughout all experiments to prevent possible adherence of PMN to inner walls with consequent artefactual selection of PMN subsets.

© 1993 Rapid Communications of Oxford Ltd

*Modulation of membrane molecules:* PMN from eight healthy donors were incubated either alone or in the presence of 1 μm chemotactic peptide formyl-methionyl-leucyl-phenylalanine (FMLP) with increasing amounts of carnitine—a kind gift of Sigma-Tau, Pomezia, Roma, Italy—(from 12.5 to 100 μg/ml in two-fold dilutions) at 37°C for 20 min with occasional shaking.

These conditions of time and temperature were chosen as they had been found suitable in inducing minimal spontaneous PMN activation while still allowing PMN to respond to appropriate stimuli. At the end of incubation time, PMN were washed free of carnitine in cold phosphate buffered saline containing 0.1% bovine serum albumin (PBS–BSA) and immediately stained with the appropriate amount of phycoerythrin-conjugated (PE) monoclonal antibody to CD11b (Dako A/S, Copenhagen, Denmark) in a single colour immunofluorescence assay. Incubation lasted 30 min and PMN were strictly maintained at +4°C to prevent further modulation of membrane molecules. Background fluorescence was determined with PE mouse IgG. PMN were finally washed in cold PBS–BSA and immediately analysed by flow cytometry.[5]

*Reactive oxygen species production:* Since PMN found in inflammatory sites are stimulated to produce reactive oxygen species (ROS),[6] the influence of carnitine (12.5 to 400 μg/ml in two-fold dilutions) on ROS production was measured by either unstimulated or phorbol myristate acetate (PMA) stimulated PMN from ten healthy donors. PMA was used at 0.1 and 1 μg/ml. PMN were loaded with the nonfluorescent fluorescein derivative dichlorofluorescein diacetate (DCHF) which is rapidly oxidized into a fluorescent compound after ROS contact and so can be measured by flow cytometry.[6] After 20 min at 37°C, PMN were washed in cold PBS–BSA, and immediately analysed by flow cytometry.

*Protection from deterioration on storage:* To explore whether carnitine was able to prolong PMN lifespan, PMN from six healthy donors were incubated at 37°C for 24 and 48 h (a condition leading to an ageing dependent metabolic impairment) in the presence of increasing amounts of carnitine (from 12.5 to 400 μg/ml in two-fold dilutions). At the end of incubation, PMN were washed and incubated with 5 μM rhodamine 123, a fluorescent probe that stains mitochondria in direct relationship to their oxidoreductive capabilities,[7] which can be regarded as a direct marker of the energy-supplying metabolic process. Incubation lasted 10 min at 37°C. PMN were then washed in cold PBS–BSA, and immediately analysed by flow cytometry.

*Flow cytometry:* Flow cytometry analyses were performed on a Becton Dickinson FACScan using log amplification for fluorescence signals. Events of interest, i.e., PMN were electronically gated using appropriate combinations of forward and side light scatter, measures of size and granularity, respectively, PMN, monocytes, lymphocytes and remnants of lysed erythrocytes each having distinct light-scattering properties. Thus, PMN can be gated for measurement of fluorescence intensity independent of the other cell types. For each experimental condition, 5000 PMN were counted. Data were processed with a Consort 30 Data Management System (Becton Dickinson). Fluorescence intensity was quantitated as the mean channel number of the fluorescence histogram distribution.[5]

*Production of TNF-α:* For measuring TNF-α production, PMN had to be purified from other peripheral blood leucocytes. This was accomplished using a standard technique including dextran sedimentation and Ficoll–Hypaque centrifugation.[4] Residual red cells were lysed by cold buffered ammonium chloride. One million PMN were incubated with a preparation of heat-killed *Staphylococcus aureus* ($2 \times 10^7$ cells) in the presence of carnitine (from 50 to 400 μg/ml in two-fold dilutions) at 37°C for 18 h. Controls included PMN alone and PMN plus carnitine but without *S. aureus*. At the end of incubation, preparations were centrifuged, the supernatant collected and assayed for TNFα content using a commercial TNF ELISA kit (Genzyme Co. Cambridge, MA, USA) according to the manufacturer's instructions. PMN from ten healthy donors were used for this assay. Data were expressed as pg/ml.

### Results

*Membrane molecule modulation:* As shown in Fig. 1A and B, carnitine alone exerted detectable effects on CD11b density although a clear dose–effect relationship was not always evident and the final result appeared to be donor dependent. However, all subjects examined showed a behaviour similar to that depicted in Fig. 1A and B. The same was true also when PMN were co-stimulated with FMLP (Fig. 1C and D). Thus, the ultimate effect of carnitine on a given PMN preparation was essentially the same irrespective of whether PMN had been primed by FMLP (Fig. 1A and C, and Fig. 1B and D).

*ROS production:* Carnitine interfered with ROS production in all subjects ($n = 10$) and its activity was fairly consistent in each individual, irrespective of the presence of PMA (Fig. 2). The final effect of the interaction between carnitine and PMN was

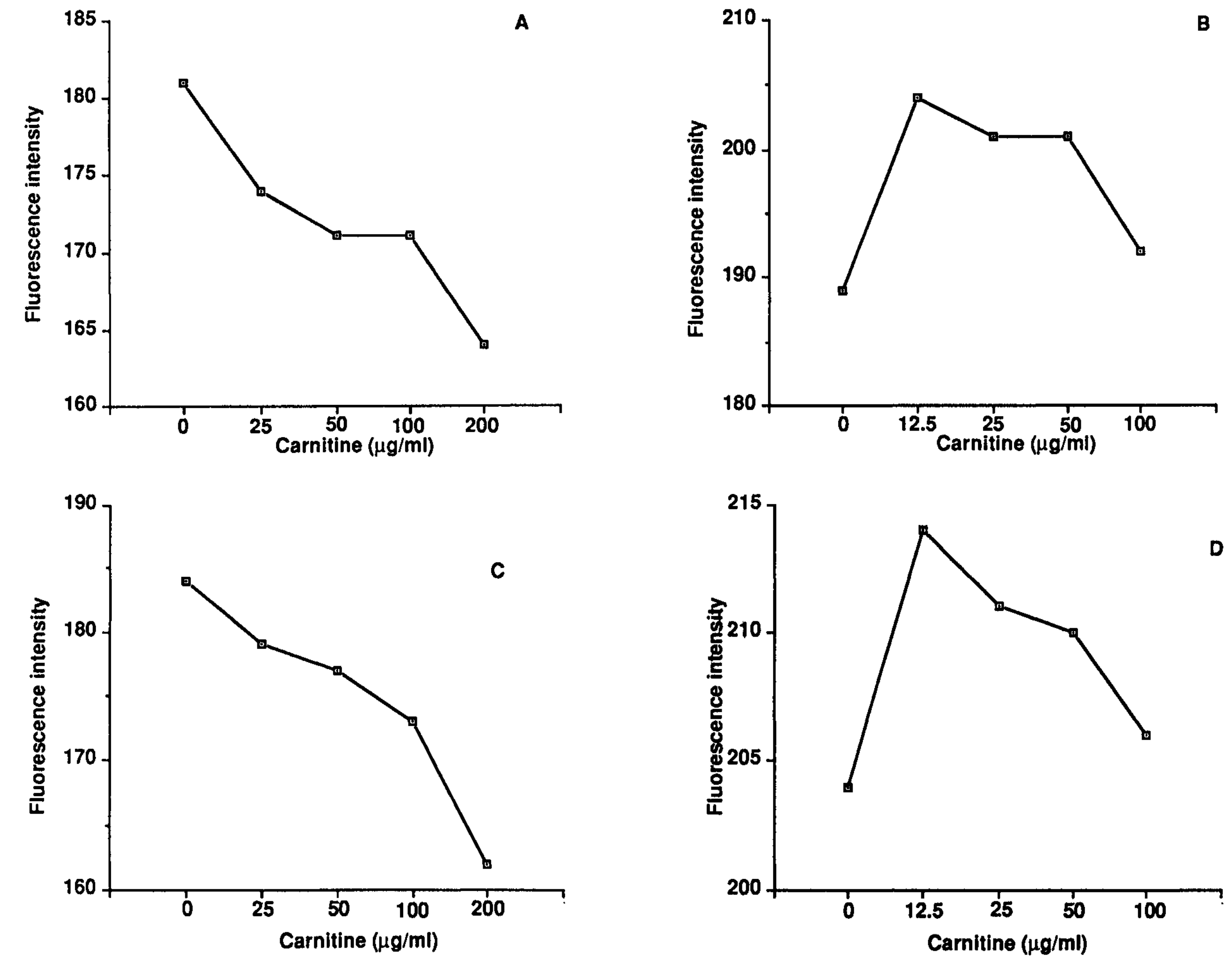

FIG. 1. Effect of incubation of PMN with carnitine (A and B) or with carnitine and 1 μM FMLP (C and D) on CD11b expression. The intensity of fluorescence (y axis) was computed from a flow cytometry generated histogram and taken as a measure of CD11b density on the PMN membrane. Sections A and C, and B and D each refer to a single individual. The results represent the two paradigmatic behaviours of all subjects examined.

donor dependent, although two paradigmatic behaviours could be observed. Low concentrations of carnitine induced a reduction followed by an increase in ROS production when PMN exhibited low basal and low PMA stimulated ROS production, whereas the opposite was true for PMN with high basal and high PMA stimulated ROS production. When PMA was used at 1 μg/ml, essentially similar results were observed (not shown).

*Protection on storage:* The effects of carnitine on PMN mitochondrial metabolism are shown in Fig. 3. After a 24 h incubation, carnitine influenced PMN from different subjects in a variable manner, although two paradigmatic behaviours could be observed (Fig. 3A and B). When PMN were incubated for 48 h, no consistent results could be obtained (not shown).

*TNFα production:* Carnitine decreased TNFα production in *S. aureus* stimulated PMN in a dose-dependent fashion. Statistical analysis (Student's test for paired samples) indicated a significant reduction in TNFα production starting from 100 μg/ml of carnitine (Fig. 4).

### Discussion

The frequent and severe infectious diseases that occur in subjects whose PMN are functionally deficient testify to the pivotal role played by PMN in the host defence against invading pathogens.[2] To accomplish their defensive activities, PMN must undergo a series of sequentially related events that lead them from the bloodstream into the inflammatory sites. Each step of this cascade of events is in turn regulated by cellular mechanisms that must be finely tuned in order to allow efficient antimicrobial activity.[2,3] In the present paper the possible effects of carnitine, an essential component of cellular energy metabolism on some of the essential steps involved in PMN functioning, namely adhesion molecule modulation, ROS production, and TNFα production and release have been investigated. Protection from deterioration on storage was also studied as a model for PMN

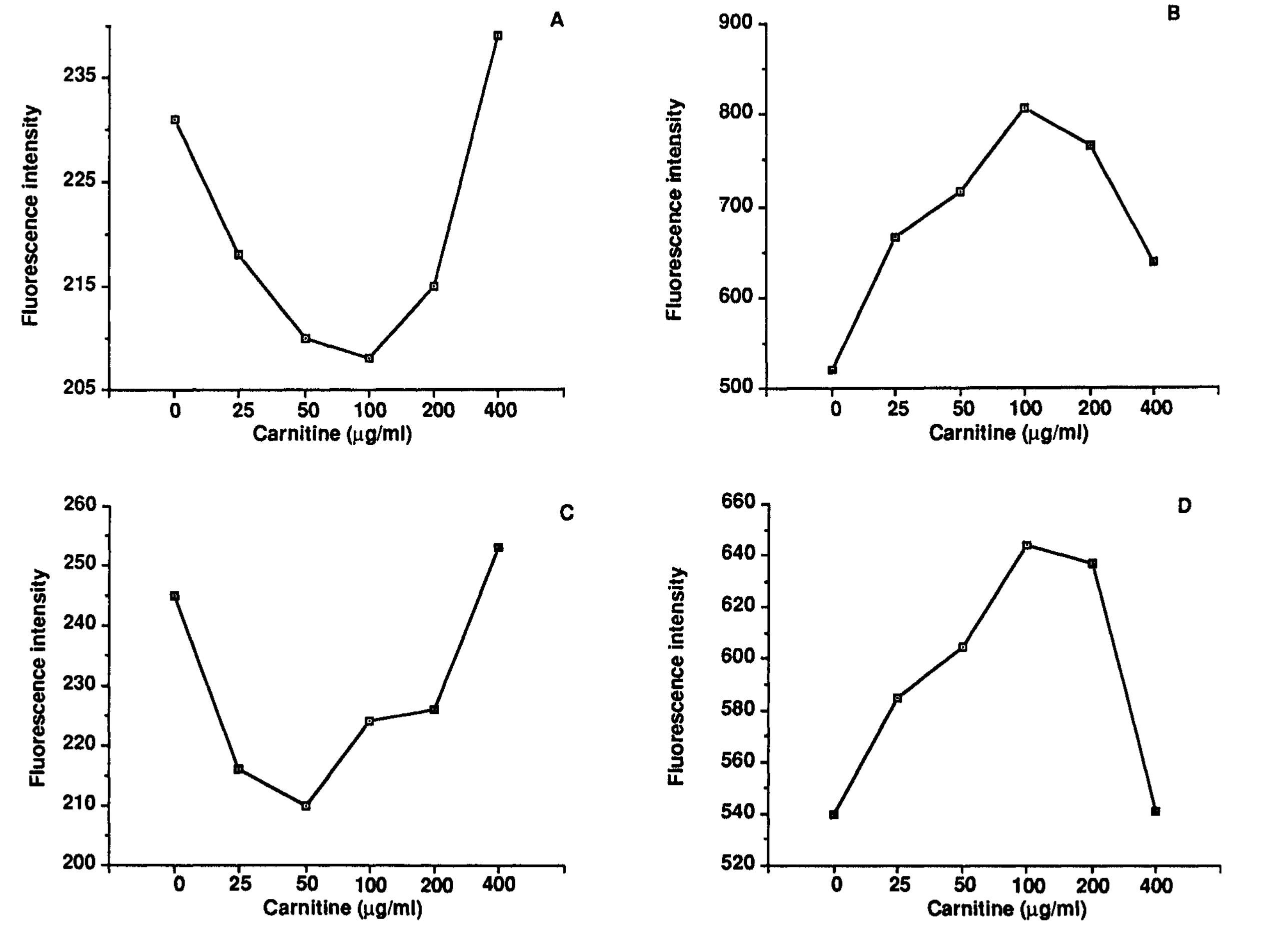

FIG. 2. Effect of incubation of PMN with carnitine (A and B) or with carnitine and 0.1 μg/ml PMA (C and D) on ROS production. The intensity of fluorescence (y axis) was computed from a flow cytometry generated histogram and taken as a measure of ROS production. Sections A and C, and B and D each refer to a single individual. The results represent the two paradigmatic behaviours of all subjects examined.

survival in tissues. The data obtained indicate that the membrane density of one of the most important adhesion molecules, beta-2-integrin CD11b,[3] is in fact modulated by carnitine. Thus, one can speculate that the interaction between this integrin and its counter-receptor on endothelial cells is under the control of carnitine. Since similar effects were observed also in FMLP primed PMN, it is conceivable that the activity of carnitine is not dependent on the induction of an activation status of PMN.

It must be noticed, however, that the effects of carnitine varied with each donor and that two main paradigmatic behaviours could be observed. This indicates that the ultimate effect of carnitine on normal resting or activated PMN is dependent upon the initial status of the cells. Similar considerations apply when considering the data obtained in ROS production and protection on storage assays: ROS are essential for an efficient killing of ingested micro-organisms while the survival of PMN and the maintenance of a satisfactory energy metabolism may be central to more distal effects, i.e., functional activities. Interestingly, gamma-interferon has been proved able to modulate the activity of PMN and counteract the decline of their functional activities induced by overnight storage.[8] This suggests that PMN metabolism can be influenced by pharmacologically active agents.

TNFα production by *S. aureus* stimulated PMN gave fairly consistent results. Carnitine was able to significantly reduce TNFα production in a dose-dependent manner starting from 100 μg/ml. This observation appears of particular interest since TNFα is an inflammatory and immunoregulatory cytokine that has been shown to play an important role in the pathogenesis of septic shock.[9] Although the major sources of TNFα are cells of the mononuclear phagocyte lineage, PMN have recently been shown to contribute to TNFα production upon appropriate stimulation.[3,10] The present observations confirm and extend those previous reports demonstrating that normal human PMN stimulated to produce and release TNFα by common bacteria can be regulated by carnitine.

To the authors' knowledge, this is the first demonstration of the capacity of carnitine to consistently influence the production of cytokines

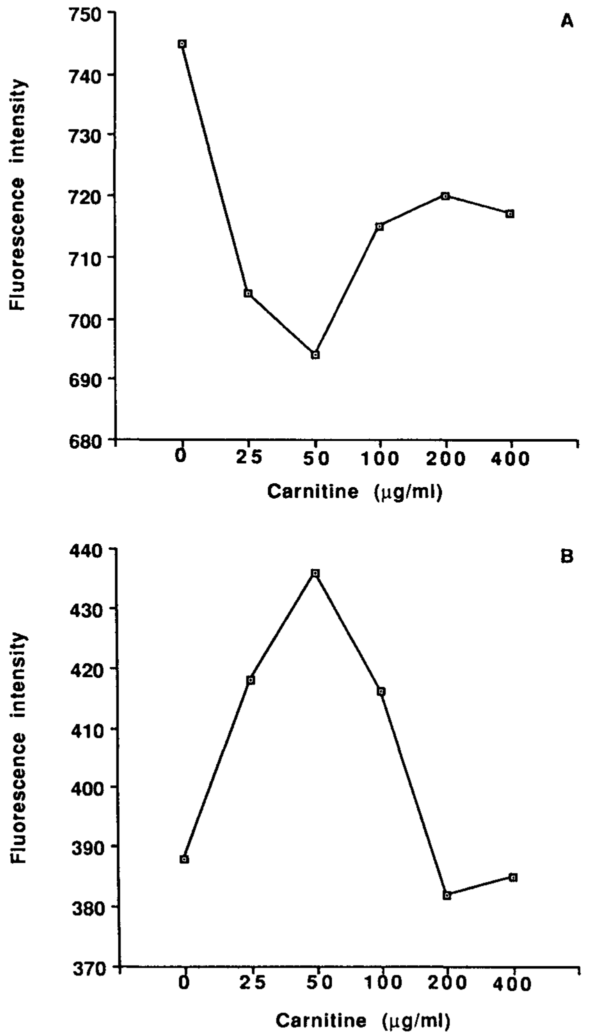

FIG. 3. Effect of incubation of PMN with carnitine on protection on storage. The intensity of fluorescence (y axis) was computed from a flow cytometry generated histogram and taken as a measure of mitochondria energy metabolism. Sections A and B each refer to a single individual. The results represent the two paradigmatic behaviours of all subjects examined.

in cells of the immune system. If substantiated by additional studies, it is conceivable that the present observation will be of relevance in clinical settings where a reduction of TNFα production is desirable.

To summarize, carnitine exerts sizeable effects on several PMN activities instrumental for the host's defence against pathogens. In addition, since a normal and finely tuned PMN functioning is essential for preventing tissue damage also in non-infection driven inflammatory situations, e.g.,

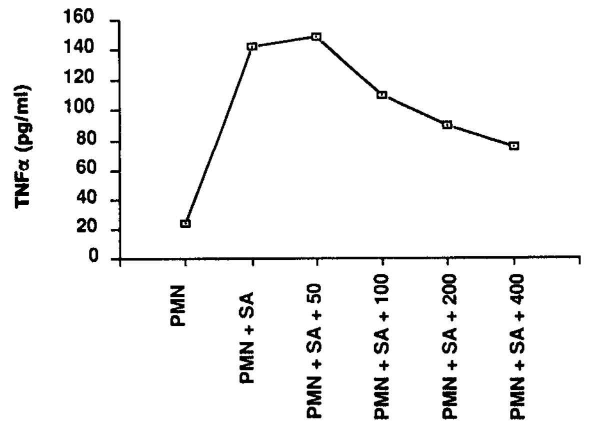

FIG. 4. Effect of incubation of PMN with carnitine on stimulation of TNFα production and release by *S. aureus*. Each data point represents the mean value obtained from ten subjects. The differences between no carnitine and carnitine 100, 200 and 400 μg/ml were $p < 0.05$, $p < 0.03$ and $p < 0.07$, respectively. SA = *S. aureus*.

after endothelial damage,[11] it is possible to speculate that carnitine may represent a useful agent for PMN regulation.

### References

1. Cress AP, Fraker PJ, Bieber LL. Carnitine and acylcarnitine levels of human peripheral blood lymphocytes and mononuclear phagocytes. *Biochim Biophys Acta* 1989; **992**: 135–139.
2. Klebanoff SJ, Clark RA. *The neutrophil: function and clinical disorders.* Amsterdam: Elsevier, 1978.
3. Lloyd AR, Oppenheim JJ. Poly's lament: the neglected role of the polymorphonuclear neutrophil in the afferent limb of the immune response. *Immunol Today* 1992; **13**: 169–171.
4. Fattorossi A, Nisini R, Le Moli S, De Petrillo G, D'Amelio R. Flow cytometric evaluation of nitro blue tetrazolium (NBT) reduction in human polymorphonuclear leukocytes. *Cytometry* 1992; **11**: 907–912.
5. Biselli R, Matricardi PM, D'Amelio R, Fattorossi A. Multiparametric flow cytometric analysis of the kinetics of surface molecule expression after polyclonal activation of human peripheral blood T lymphocytes. *Scand J Immunol* 1992; **35**: 439–447.
6. Barsotti P, Biselli R, Nisini R, Pecci G, Oliva C, D'Amelio R, Fattorossi A. Effects of hypobaric hypoxia on the number and metabolic activity of circulating polymorphonuclear granulocyte in acclimatized and non acclimatized rats. In: Ueda G, Reeves JT, Sekiguchi M, eds. *High altitude medicine.* Matsumoto: Shinshu University Press, 1992; 59–63.
7. Darzynkiewicz Z, Staiano-Coico L, Melamed MR. Increased mitochondrial uptake of rhodamine 123 during lymphocyte stimulation. *Proc Natl Acad Sci USA* 1981; **78**: 2383–2387.
8. Seymour B, Klebanoff J, Olszowski S, Van Voorhis W, Ledbetter JA, Waltersdorph AM, Schlechte KG. Effects of gamma-interferon on human neutrophils; protection from deterioration on storage. *Blood* 1992; **6**: 225–234.
9. Cerami A. Inflammatory cytokines. *Clin Immunol Immunopathol* 1992; **62**: S3–S10.
10. Mandy Y, Endrész V, Krenàcs L, Régely K, Degré M, Bélèdi I. Tumour necrosis factor production by human granulocytes. *Inf Anch Allergy Appl Immunol* 1991; **96**: 102–106.
11. Jagels MA, Hugli TE. Neutrophil chemotactic factors promote leukocytosis. A common mechanism for cellular recruitment from bone marrow. *Immunol J* 1992; **148**: 1119–1128.

ACKNOWLEDGEMENTS. This work was supported by Cattedra Malattie Infettive, Universitá di L'Aquila, Italy.
